# Preparation of Metallic Zr from ZrO_2_ via Carbothermal and Electrochemical Reduction in Molten Salts

**DOI:** 10.3390/ma18112634

**Published:** 2025-06-04

**Authors:** Wenchen Song, Xu Chen, Yanhong Jia, Mingshuai Yang, Guoan Ye, Fuxing Zhu

**Affiliations:** 1 Department of Radiochemistry, China Institute of Atomic Energy, Beijing 102413, China; 2 Panxi Institute of Vanadium and Titanium Inspection and Testing, Panzhihua 617000, China; 3 State Key Laboratory of Vanadium and Titanium Resources Comprehensive Utilization, Pangang Group Research Institute Co., Ltd., Panzhihua 617000, China

**Keywords:** metallic zirconium, oxycarbide anode, carbothermal reduction, molten salt electrolysis, solid solution

## Abstract

Zirconium, a critical rare metal with exceptional corrosion resistance and nuclear applications, is conventionally produced via the energy-intensive Kroll process. The electrolysis of ZrC_x_O_y_ soluble anodes has been extensively investigated due to its advantages in having a short process flow and resulting in high-quality products. In particular, during the electrolysis of zirconium oxycarbide with a C:O molar ratio of 1:1, gaseous CO can be released, and no residual anodes are generated, which is extremely appealing. In this regard, this paper explores the feasibility of preparing zirconium metal through high-temperature vacuum reduction to produce zirconium oxycarbide using ZrO_2_ as the raw material, followed by direct molten-salt electrolysis. Firstly, the reduction products were characterized using an X-ray diffractometer (XRD) and a scanning electron microscope (SEM). The results showed that under a vacuum of <10 Pa at 1750 °C, the reduction products mainly consisted of ZrC_x_O_y_ and a small amount of ZrO_2_, and they exhibited good electrical conductivity (0.0169 Ω·cm). Subsequently, the cyclic voltammetry test results of the reduction products revealed the reversible redox behavior of ZrC_x_O_y_. There were characteristic oxidation peaks at −0.53 V and −0.01 V (vs. Pt), corresponding to the formation of Zr^2+^ and Zr^4+^, respectively, and a reduction peak at −1.51 V, indicating the conversion from Zr^2+^ to Zr. Finally, *β*-zirconium metal with a purity of 99.2 ± 0.3 wt.% was obtained through potentiostatic electrolysis, and its quality met the R60704 grade specified in ASTM B551-12 (2021). This study offers a novel approach for the short-flow preparation of zirconium metal, which is conducive to expanding its applications.

## 1. Introduction

Zirconium is an important rare metal with excellent corrosion resistance, good mechanical properties, and a low thermal neutron absorption cross section; this material is widely used in the aerospace, nuclear, and chemical industries [1,2]. Global zirconium resources are abundant, but mainly in the form of zircon (ZrSiO_4_), and zircon is often symbiotic with other minerals, which makes it difficult to substantially increase zirconium extraction [3,4]. Currently, zirconium metal is mainly prepared industrially through the magnesium thermal reduction of ZrCl_4_ (Kroll process), but this process is time consuming, necessitates high energy consumption, and is expensive [5,6]. Therefore, compared with thermal reduction processes, molten salt electrolysis has received more attention because of its time efficiency and the good quality of the obtained products [7,8].

According to the different raw materials used in electrolysis, Zr metal preparation can be divided into zirconium halide and oxide electrolysis process; the former is mainly used in the preparation of zirconium metal via the direct molten salt electrolysis of halide raw materials, such as ZrCl_4_, and K_2_ZrF_6_, but it is still poorly efficient and expensive [9,10]; the latter mainly uses ZrO_2_ as the raw material to prepare zirconium metal via molten salt electrolysis. Li et al. prepared Zr–Mg alloys via the electrochemical deoxidization of ZrO_2_–MgO, using the FFC Cambridge process. Unfortunately, this process has also low efficiency, and the obtained products contain excessive oxygen impurities [11,12]. A new process for the electrolytic preparation of zirconium metal was proposed by Shang et al. [13]. They prepared a conductive ZrC_x_O_y_ anode via electrochemical reduction using a ZrO_2_ and C mixture as the cathode and graphite as the anode; they then electrolyzed ZrC_x_O_y_ to obtain zirconium metal, while a carbon steel rod was used as the cathode. The zirconium metal obtained via this process has high purity, and the electrolysis process is stable, but the preparation of ZrC_x_O_y_ soluble anodes is affected by a low efficiency and poor material uniformity. Actually, ZrO_2_ can be reduced to ZrC_x_O_y_ using ZrC and Zr above 1600 °C [14]. Li et al. systematically investigated the influence of different carbon-to-ZrO_2_ ratios on the reduction product ZrC_x_O_y_ at 1600 °C under argon protection. Their findings indicated that when the molar ratio of ZrO_2_ to C is 1:2, the reduction products are ZrC_x_O_y_ and ZrO_2_, rather than the target product ZrC_0.5_O_0.5_. When the molar ratio of ZrO_2_ to C is 1:2.6, a pure ZrC_0.79_O_0.21_ phase can be obtained. Using this as the raw material, they successfully prepared zirconium metal via electrolysis [15]. However, a certain amount of residual carbon is generated at the anode, which can reduce electrolysis efficiency and contaminate the product quality as the electrolysis progresses. Jiao et al. demonstrated that when the carbon-to-oxygen molar ratio in titanium oxycarbide (TiC_x_O_1−x_) is 1:1 (x = 0.5), electrolysis with a soluble anode eliminates residual anode formation and enables metallic titanium deposition on the cathode [16]. Inspired by this, it is hypothesized that ZrC_0.5_O_0.5_ electrolysis products may also produce minimal residual anodes. Nevertheless, it remains unclear whether ZrO_2_ can be directly carbothermally reduced to ZrC_0.5_O_0.5_ at higher temperatures and under vacuum conditions. Additionally, it is unknown whether the reduction products can be directly used in molten-salt electrolysis to prepare zirconium metal.

This study proposes a carbothermal and electrochemical reduction process for the preparation of zirconium metal, focusing on the electrochemical dissolution and deposition of ZrC_x_O_y_ prepared through the carbothermal reduction of ZrO_2_ and the ZrC_x_O_y_ soluble anode. The results demonstrate that ZrO_2_ can be reduced to conductive ZrC_x_O_y_ through reaction with graphite at high temperatures, and a metallic Zr was electrolyzed from this ZrC_x_O_y_ as an anode in molten salts.

## 2. Materials and Methods

### 2.1. Carbothermal Reduction of ZrO_2_

To gain in-depth insights into the reduction process of ZrO_2_ by carbothermal reduction, the thermodynamic parameter conditions for the reduction of zirconium oxycarbide were calculated. Although direct thermodynamic data for ZrC_0.5_O_0.5_ are lacking, the formation of an infinite solid solution between ZrO and ZrC enables us to deduce the synthesis conditions of ZrC_0.5_O_0.5_ by analyzing the reduction of ZrO_2_ to these two phases [14,17]. By using HSC Chemistry 6.0 software, thermodynamic calculations for the reduction of ZrO_2_ resulted in the following reaction:

2ZrO_2_ + 4C→ZrC + ZrO + 3CO(g)       ΔG^θ^ = 1518.1 − 0.689*T* (1)

Over the 0–2000 °C range, the positive standard Gibbs free energy of Reaction 1 indicates non-spontaneity under ambient pressure. Under a vacuum, the Gibbs free energy is modified to account for CO partial pressure [18]:

ΔG = ΔG^θ^ + R*T* ln(*P*_CO_/*P*^0^)^3^ = 1518.1 − 0.689*T* + 0.02492*T* ln(*P*_CO_/*P*^0^) (2)

**Figure 1:**
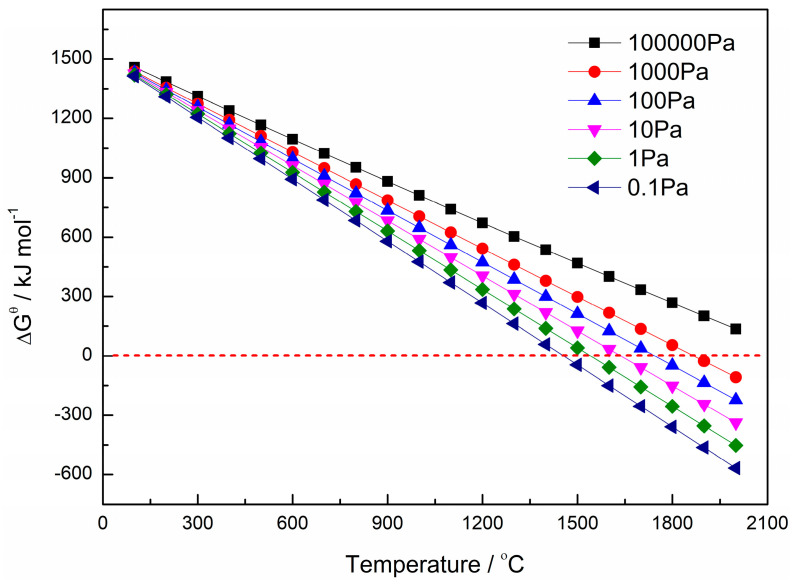
Temperature-dependent Gibbs free energy at different vacuum levels.

Figure 1 illustrates the temperature-dependent Gibbs free energy at varying vacuum levels. Increasing temperature lowers ΔG^θ^, with vacuum enhancement (i.e., reduced total pressure) accelerating this decline. At 0.1 Pa, spontaneity is achieved at 1500 °C. Balancing furnace operational limits, the optimized reduction parameters were set as 1750 °C, 5 h, and a vacuum level <10 Pa.

Under the aforementioned reduction conditions, specific amounts of ZrO_2_ powder (AR, Shanghai Macklin Biochemical Technology Co., Shanghai, China) and graphite powder (<45 µm, Qingdao Tianheda graphite Co., Ltd., Qingdao, China) were first weighed and placed in a three-dimensional mixer (SYH-5, Changzhou Yineng drying equipment Co., Ltd., Changzhou, China) with ZrO_2_:C molar ratio of 1:2. After 4 h of mixing, 3 wt.% polyvinyl alcohol binder was added, and mixing was continued for 2 h. Then, about 50 g of the mixture was placed in a 30 mm diameter grinding tool under a four-column hydraulic press (Y32-250T, Tengzhou Hairun Machine Tool Co., Ltd., Tengzhou, China); a pressure of 20 MPa was applied for 20 s to obtain the required block. This column was then placed in a 120 °C oven for drying for 12 h and later transferred to a vacuum furnace (JVIM, Shenyang Jinyan New Material Preparation Technology Co., Ltd., Shenyang, China) for carbothermal reduction. After the reduction, the reduction products were taken out of the furnace for weighing and were characterized using X-ray diffraction (XRD, X’pert PRO, PANalytical, Almelo, The Netherlands) and scanning electron microscopy in combination with an energy-dispersive spectrometer (SEM-EDS, SIGMA 500, Carl Zeiss AG, Jena, Germany).

### 2.2. Electrochemical Measurements and Electrodeposition

The electrochemical behavior of the reduced products was characterized by cyclic voltammetry (CV) in NaCl-KCl (equimolar ratio) molten salt under the protection of argon gas using an electrochemical workstation (PARSTAT 4000A, AMETEK, Inc., Berwyn, IL, USA) connected to a three-electrode system. ZrC_x_O_y_ was embedded in a small hole of a molybdenum rod with a diameter of 4 mm (hole diameter of 2 mm), which was used as the working electrode. A platinum rod with a diameter of 4 mm and a graphite rod with a diameter of 4 mm were used as the quasi-reference and counter electrodes, respectively.

After the electrochemical measurements, the hollow reduction product was connected to a graphite rod, which was then connected to the positive terminal of a power amplifier (BOP 20-20M, KEPCO, Inc., New York, NY, USA), while the negative terminal was connected to a molybdenum rod with a diameter of 8 mm. Constant-potential electrolysis was performed at a reduction potential of −1.6 V (vs. Pt) to study the dissolution characteristics of ZrC_x_O_y_. After electrolysis, the cathode and anode products were removed from the molten salt and cooled to room temperature in argon; they were then washed in 0.5 wt.% hydrochloric acid to remove the electrolyte trapped within the cathode and anode products; the hydrochloric acid was subsequently washed off with deionized water to obtain the cathodic products and anodic residue. The cathode products were also characterized via XRD and SEM. In addition, inductively coupled plasma emission spectrometry (ICP-AES, NexION 300D, PerkinElmer, Waltham, MA, USA) was employed to analyze components such as Zr, Fe, and Cr in the deposited products [19,20]. For the determination of O, N, and H, an oxygen, nitrogen, and hydrogen analyzer (Leco ONH 836, Saint Joseph, MI, USA) was utilized.

## 3. Results and Discussion

### 3.1. Carbothermal Reduction

**Figure 2:**
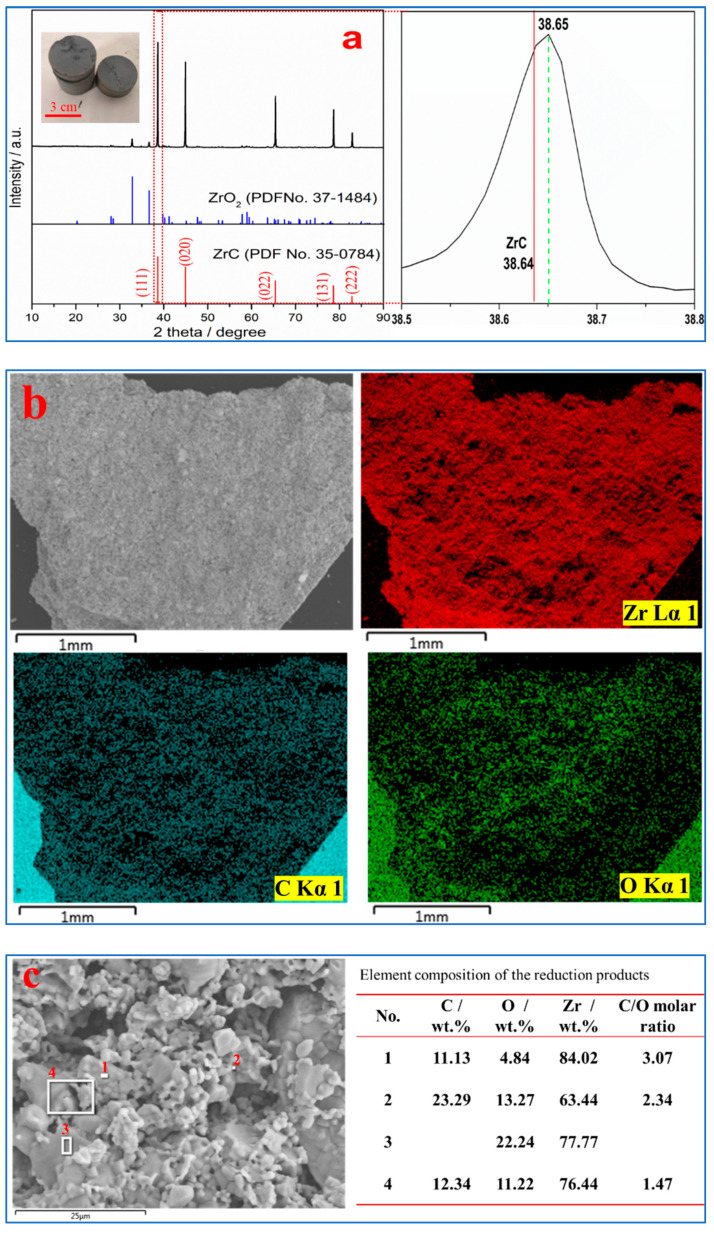
XRD patterns (**a**), and SEM–EDS results (**b**,**c**) of the reduction products.

Figure 2a shows that the reduced product exhibits a black-colored, dense morphology. The main diffraction peaks of the reduced product are close to those of ZrC, but a local magnification of the (111) plane reveals that the diffraction peak of the reduced product is shifted by +0.01° compared to pure ZrC. In face-centered cubic ZrC, carbon atoms can be substituted by oxygen atoms to form ZrCₓO_y_ solid solutions, which causes a positive shift in diffraction peaks—this phenomenon is consistent with the behavior of TiCₓO_y_, whose diffraction peaks lie between those of TiC and TiO [21]. Notably, the reduced product in Figure 2a still contains trace amounts of ZrO_2_. Given that low-valent zirconium oxides and ZrCₓO_y_ with high oxygen content are unstable phases [22,23], and combining with the preferential formation of TiC during TiO_2_ reduction, the reduction pathway of ZrO_2_ is proposed as ZrO_2_→ZrC→ZrC_x_O_y_. Additionally, compared with the results of Li et al., the absence of diffraction peaks from residual carbon and the lower intensity of ZrO_2_ peaks in the reduced product—achieved via vacuum and higher-temperature reduction—indicate a more complete reduction reaction [15]. In addition, Figure 2b,c show that carbon (C) and oxygen (O) are generally uniformly distributed in zirconium oxycarbide, although localized regions with unreduced ZrO_2_ persist. The C/O molar ratios vary across the product: in Figure 2c, the ratio is 3.07 at position 1 and 1.47 at position 4, indicating local compositional heterogeneity. Notably, particles with higher C/O ratios exhibit finer sizes, while ZrO_2_ particles are coarser; regions adjacent to ZrO_2_ show lower C/O ratios, further supporting the reaction between ZrC and ZrO_2_ to form ZrC_x_O_y_. These findings suggest that ZrO_2_ is preferentially reduced to ZrC, which then reduces surrounding ZrO_2_ to ZrC_x_O_y_. However, the formation of large pores during reduction hinders the further conversion of ZrO_2_ to ZrC_x_O_y_, resulting in a C/O molar ratio >1 in zirconium oxycarbide.

Table 1 shows that the weight loss rate of the reduced products is 26.91%, and the reduction rate reaches 94.16% (while the theoretical weight loss rate is 28.58%). The resistivity of the reduction products (0.0169 Ω cm) is lower than that before reduction (0.0440 Ω cm), indicating that the reduction products have good electrical conductivity and are suitable for electrolysis as soluble anodes.

### 3.2. Electrochemical Behavior of Zirconium Oxycarbide

**Figure 3:**
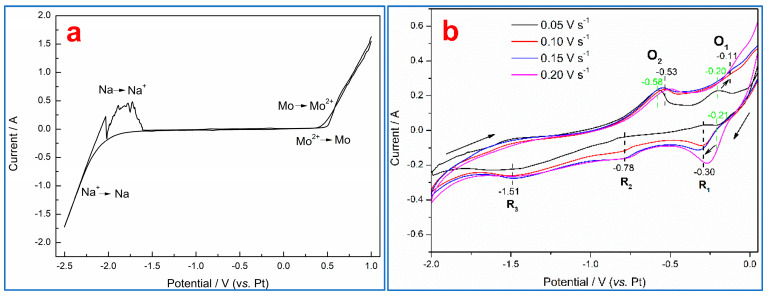
CV curves obtained for the Mo electrode (**a**) and ZrC_x_O_y_ electrode (**b**) in the KCl–NaCl melt at different scan rates.

Figure 3 shows the CV curves obtained for the Mo and ZrC_x_O_y_ electrodes in the KCl–NaCl melt at 750 °C. No other electrochemical signals are observed in the NaCl–KCl molten salts except for two pairs of redox peaks corresponding to Na^+^→Na and Mo^2+^→Mo at −2.2 V (vs. Pt) and 0.5 V (vs. Pt), respectively [21]. However, when using a ZrC_x_O_y_ working electrode, two prominent oxidation peaks (O_1_ and O_2_) emerge at −0.20 V and −0.53 V (vs. Pt) during the anodic scan, accompanied by three reduction peaks (R_1_, R_2_, and R_3_) at −0.30 V, −0.78 V, and −1.51 V (vs. Pt) during the cathodic scan. According to Li et al., the oxidation of Zr^3+^ to Zr^4+^ occurs at approximately −0.20 V (vs. Pt) [15]; thus, O_1_/R_1_ can be assigned to the Zr^3+^/Zr^4+^ redox couple. Notably, the reduction potential of R_1_ is ~0.2 V higher than reported by Li et al. [15]. This positive shift arises from the rapid diffusion and dilution of dissolved Zr ions during the anodic scan, as the initial electrolyte contains no Zr species. Based on Shang et al., the potential difference between Zr^4+^/Zr^3+^ and Zr^4+^/Zr (or Zr^+^/Zr) is ~1.2 V [13]. Therefore, R_3_ is attributed to the reduction of Zr^4+^ (or Zr^+^) to metallic Zr. The absence of a corresponding oxidation peak for R_3_ is likely due to the large electrode area (4 mm diameter Mo rod embedded with ZrC_x_O_y_), which suppresses the reverse oxidation process. The potential difference between O_1_ and O_2_ is 0.38 V, significantly smaller than the expected 0.6 V for Zr^2+^→Zr^4+^ oxidation [15]. This suggests that O_2_ corresponds to the oxidation of ZrC_x_O_y_ to Zr^3+^, with R_2_ as the complementary reduction peak. Considering that CO is the oxidation byproduct of ZrC_x_O_y_ and the molar ratio of C to O is 1:1, as reported in reference [24], the electrode reactions during electrolysis are proposed as follows:

Anode: ZrC_0.5_O_0.5_ → Zr^3+^ + 0.5CO(g) + 3e^−^ (3)

Cathode: Zr^3+^ + 3e^−^ → Zr (4)

Overall reaction: ZrC_0.5_O_0.5_ →Zr + 0.5CO(g) (5)

The above results indicate that no residual anode is generated when the C/O molar ratio in ZrC_x_O_y_ is 1:1, whereas residual carbon forms when the C/O ratio exceeds 1:1. Combined with the findings in Figure 1, this residual carbon can react with unreduced ZrO_2_ via Reaction 6 to produce metallic zirconium and CO, suggesting that during the reduction process under a carbon-to-oxygen ratio of 1:1, ZrC_x_O_y_ with a C/O molar ratio greater than 1:1 is formed. Although molten salt electrolysis under these conditions produces residual carbon, this residual carbon can further react with unreduced ZrO_2_ through electrolysis to form metallic zirconium and CO, thereby eliminating residual anode formation.

ZrO_2_ + 2C → Zr + 2CO(g) (6)

Additionally, Figure 3b demonstrates that increasing the scan rate from 0.05 to 0.20 V s⁻¹ causes the oxidation potential of ZrC_x_O_y_ to shift positively from −0.58 V to −0.53 V (vs. Pt), while the reduction potential shifts negatively. This behavior confirms the irreversible nature of the ZrC_x_O_y_ dissolution and reduction processes [25,26].

**Figure 4:**
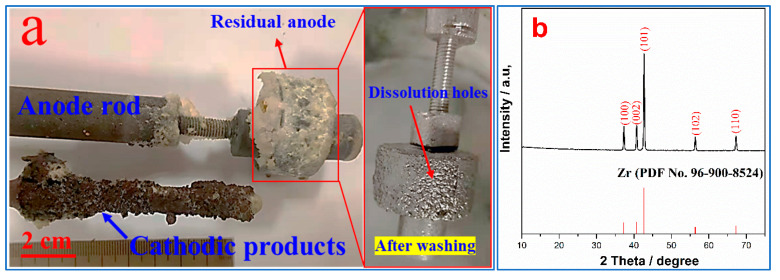

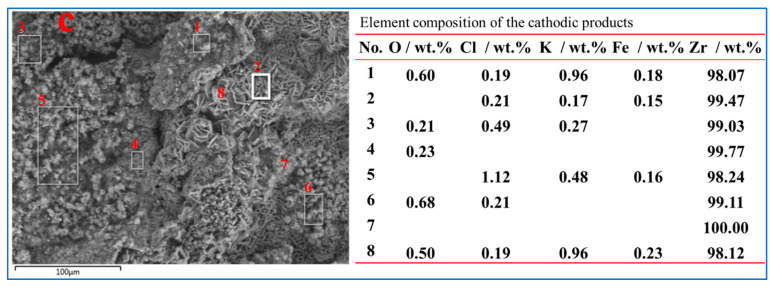
The appearance of the electrolytic products (**a**), XRD patterns (**b**) and SEM–EDS results (**c**) of the cathodic products.

The appearance of the cathode and anode products after electrolysis at −1.6 V (vs. Pt) for 3 h as well as the corresponding XRD patterns and SEM image of the cathodic products after washing are shown in Figure 4. The figure shows that the cathodic product wrapped by the electrolyte has been deposited on the Mo rod, while the anode has been wrapped by the electrolyte without undergoing dissolution. However, the residual anode after washing shows obvious dissolution traces and holes (Figure 4a), and the anode dissolution rate after washing and drying is 7.86%, which further demonstrates that ZrC_x_O_y_ can be dissolved in the NaCl–KCl molten salts. The XRD results of the washed cathodic products indicate that the deposited product is pure *β*-phase metallic zirconium (Figure 4b), which is largely consistent with the findings of Li et al. [15]. The SEM–EDS results in Figure 4c show that the cathodic product consists of fine flakes and agglomerates of particulate matter, containing small amounts of impurity elements such as O, Cl, K, and Fe. The presence of Cl, K, and O might be attributed to hydrolysis during the washing process and residual electrolyte, while the Fe impurity could result from contamination by the cathode material.

The results presented in Table 2 indicate that the purity of the electrolytically obtained zirconium metal reaches 99.2 ± 0.3 wt.%. Impurity elements such as Fe + Cr, H, O, C, and N fully meet the requirements of the R60704 grade in the American standard ASTM B551-12 (2021) [27]. Considering that the theoretical molar ratio of C to O in ZrC_x_O_y_ is 1:1, no residual carbon is generated during the electrolysis process. This results in a low carbon content in the products of the electrolysis. Although increasing the carbon-to-oxygen ratio can allow for the production of pure ZrC_x_O_y_, it also causes carbon contamination in the products. Therefore, maintaining the theoretical ratio enhances the quality of the zirconium metal. Furthermore, the continuous and stable operation of the electrolysis can be ensured by regularly removing undissolved ZrO_2_ from the anode surface with a scraper or other means. Overall, this powder can be directly used as a raw material for powder metallurgy, which can significantly reduce the cutting losses during the processing of zirconium-based materials. This circumvents the preparation of ZrCl_4_ and the use of expensive magnesium metal reductant in the production of zirconium by the Kroll process.

The above-mentioned research shows that ZrC_x_O_y_ can be synthesized from ZrO_2_ through vacuum carbothermal reduction. Subsequently, zirconium metal products can be fabricated by directly conducting molten-salt electrolysis with ZrC_x_O_y_ as a soluble anode. Compared with the direct electro-deoxidation of ZrO_2_ for zirconium metal production, this method is more efficient and yields higher-quality products, thus showing promising application prospects [11].

## 4. Conclusions

This study presents a novel carbothermal–electrolytic route for zirconium production, achieving direct conversion of ZrO_2_ to high-purity metal via a conductive oxycarbide intermediate. Key advancements include the following:

Thermodynamically optimized carbothermal reduction at 1750 °C/<10 Pa, yielding ZrC_x_O_y_ with ideal electrical conductivity (0.0169 Ω·cm).

Electrochemical validation of ZrC_x_O_y_ as a soluble anode, enabling reversible Zr^2+^/Zr^4+^ redox cycles and efficient metal deposition at −1.6 V (vs. Pt).

The production of high-purity zirconium, achieving a purity of 99.2 ± 0.3 wt.%, is characterized by the formation of fine flakes and agglomerates of particulate matter. This method circumvents the chlorination steps, thereby reducing the complexity of the production process.

This integrated approach paves the way for sustainable zirconium metallurgy, with potential for scaling to industrial capacities and extending to other refractory metals via oxycarbide intermediates.

## Figures and Tables

**Table 1:** Weight loss rate and electrical resistivity of the furnace burden before and after carbothermal reduction.

	Weight Loss Rate/%	Electrical Resistivity/Ω cm
Before reduction	/	0.0440
After reduction	26.91	0.0169

**Table 2:** Chemical compositions of Zr metal after washing.

Batch Number	Zr/wt.%	Fe + Cr/wt.%	H/wt.%	O/wt.%	C/wt.%	N/wt.%
No. 1	99.5	0.18	0.002	0.13	0.021	0.012
No. 2	99.0	0.31	0.004	0.19	0.018	0.024
No. 3	99.1	0.27	0.003	0.17	0.024	0.019
Average	99.2 ± 0.3	0.25 ± 0.07	0.003 ± 0.001	0.16 ± 0.03	0.021 ± 0.003	0.018 ± 0.06
R60704	≥97.5	0.2 to 0.4	≤0.005	≤0.18	≤0.05	≤0.025

## Data Availability

The original contributions presented in this study are included in the article. Further inquiries can be directed to the corresponding author.

## References

[1] Birch R.M., Douglas J.O., Britton T.B. (2023). Characterization of local deformation around hydrides in Zircaloy-4 using conventional and high angular resolution electron backscatter diffraction. Mater. Charact..

[2] Xu J., Li H., Zhao X., Wu J., Zhao B., Zhao H., Wu J., Zhang Y., Liu C. (2022). Zirconium based neutron absorption material with outstanding corrosion resistance and mechanical properties. J. Nucl. Mater..

[3] Perks C., Mudd G. (2019). Titanium, zirconium resources and production: A state of the art literature review. Ore Geol. Rev..

[4] Zeng Y., Liang F., Liu J., Zhang J., Zhang H., Zhang S. (2018). Highly Efficient and Low-Temperature Preparation of Plate-Like ZrB_2_-SiC Powders by a Molten-Salt and Microwave-Modified Boro/Carbothermal Reduction Method. Materials.

[5] Park K., Nersisyan H.H., Lee H., Hong S.-I., Cho N.-C., Lee J.-H. (2012). Low-temperature synthesis of zirconium metal using ZrCl_4_–2Mg reactive mixtures. Int. J. Refract. Met. H.

[6] Li S., Che Y., Shu Y., He J., Song J., Yang B. (2021). Review—Preparation of Zirconium Metal by Electrolysis. J. Electrochem. Soc..

[7] Li M., Liu C., Ting A., Xiao C. (2023). A review on the extraction and recovery of critical metals using molten salt electrolysis. J. Environ. Chem. Eng..

[8] Xi X., Feng M., Zhang L., Nie Z.-R. (2020). Applications of molten salt and progress of molten salt electrolysis in secondary metal resource recovery. Int. J. Miner. Metall. Mater..

[9] Zhang X., Huang J., Min D., Wen T., Shi Z., Yang S. (2022). Mechanism for electrochemical reduction of Zr(IV) in molten NaCl–KCl. Int. J. Hydrogen Energy.

[10] Li S., Che Y., Song J., Shu Y., He J., Xu B., Yang B. (2021). Preparation of zirconium metal through electrolysis of zirconium oxycarbonitride anode. Sep. Purif. Technol..

[11] Li N., Peng Y., Chen Z., Xiong W., Sun J., Chen Y., Zhang P., Liu M., Li S. (2021). Preparation of Mg-Zr alloys through direct electro-deoxidation of MgO-ZrO_2_ in CaCl_2_-NaCl molten salt. Electrochim. Acta.

[12] Abdelkader A., Daher A., Abdelkareem R.A., El-Kashif E. (2007). Preparation of Zirconium Metal by the Electrochemical Reduction of Zirconium Oxide. Metall. Mater. Trans. B.

[13] Shang X., Li S., Che Y., Shu Y., He J., Song J. (2021). Novel extraction of Zr based on an in-situ preparation of ZrC_x_O_y_. Sep. Purif. Technol..

[14] Liu H., Man Z., Liu J., Wang X.-G., Zhang G.-J. (2017). Solid solution and densification behavior of zirconium oxycarbide (ZrC_x_O_y_) ceramics via doping ZrO_2_ and Zr in ZrC. J. Alloys Compd..

[15] Li S., Che Y., Song J., Shu Y., Xu B., He J., Yang B. (2021). Electrolytic Preparation of Zirconium Metal from a Consumable Zirconium Oxycarbide Anode. Metall. Mater. Trans. B.

[16] Jiao S., Zhu H. (2006). Novel metallurgical process for titanium production. J. Mater. Res..

[17] Katea S.N., Riekehr L., Westin G. (2021). Synthesis of nano-phase ZrC by carbothermal reduction using a ZrO_2_–carbon nano-composite. J. Eur. Ceram. Soc..

[18] Wang J., Jia C., Li C., Peng X.-L., Zhang L.-H., Liu J.-Y. (2019). Thermodynamic Properties for Carbon Dioxide. ACS Omega.

[19] Hamm D., Ogle K., Olsson C.-O.A., Weber S., Landolt D. (2002). Passivation of Fe–Cr alloys studied with ICP-AES and EQCM. Corros. Sci..

[20] Mehdi N., Pouyan S. (2009). A simple method for determining Hf in Zr and Zr alloys by ICP-AES. Nucl. Sci. Tech..

[21] Long W., Gao F., Wang D., Zou B., Wang X., Wang Y., Zhan F., Wei Y. (2022). Forming thermodynamics, structure, and electrical conductivity of TiC_x_O_y_ compounds fabricated through the carbothermal reduction process. J. Alloys Compd..

[22] Pham D., Gai F., Rochester J., Dycus J.H., LeBeau J.M., Manga V.R., Corral E.L. (2023). Thermodynamic assessment within the Zr–B–C–O quaternary system. J. Am. Ceram. Soc..

[23] Efaw C.M., Vandegrift J.L., Reynolds M., McMurdie S., Jaques B.J., Hu H., Xiong H., Hurley M.F. (2019). Characterization of zirconium oxides part I: Raman mapping and spectral feature analysis. Nucl. Mater. Energy.

[24] Li S., Lv Z., He J., Song J. (2025). Anodic dissolution of consumable ZrC_x_O_y_ and cathodic deposition of zirconium in NaCl-KCl based melts. J. Mol. Liq..

[25] Zhang K., Xu L., Xia D., Meng J., Zhao Z. (2023). A fundamental study on indium electrochemistry in molten NaCl-KCl eutectic: Toward new technology development for indium secondary resources recycling. Ionics.

[26] Wang Z., Tada E., Nishikata A. (2015). In Situ Analysis of Scan Rate Effects on Pt Dissolution Under Potential Cycling Using a Channel Flow Double Electrode. Electrocatalysis.

[27] (2021). Standard Specification for Zirconium and Zirconium Alloy Strip, Sheet, and Plate.

